# Impact of Subthreshold Micropulse Laser on the Vascular Network in Diabetic Macular Edema: An Optical Coherence Tomography Angiography Study

**DOI:** 10.3390/biomedicines13051194

**Published:** 2025-05-14

**Authors:** Barbara Sabal, Edward Wylęgała, Sławomir Teper

**Affiliations:** 1 Chair and Clinical Department of Ophthalmology, Faculty of Medical Sciences in Zabrze, Medical University of Silesia, 40-752 Katowice, Poland; 2 Department of Ophthalmology, John Paul II Municipal Hospital, 35-241 Rzeszow, Poland; 3 Scientific Research Facility, Branch in Bielsko-Biala, Medical University of Silesia, 40-752 Katowice, Poland

**Keywords:** diabetic macular edema, diabetic retinopathy, subthreshold micropulse laser, optical coherence tomography angiography, OCTAVA, foveal avascular zone, microaneurysms

## Abstract

**Objectives**: To evaluate the short- and long-term effects of subthreshold micropulse laser (SMPL) treatment on the microvascular network in diabetic macular edema (DME). **Methods**: This 12-month prospective study included 67 eyes (67 patients) with mild DME and good best-corrected visual acuity (BCVA), randomized into SMPL (33 eyes) or sham (34 eyes) groups. Assessments were performed at baseline (T1), 3 months (T2), and 12 months (T3). Optical coherence tomography (OCT) and OCT angiography (OCTA) were used to measure central retinal thickness (CRT), macular thickness (MT), macular volume (MV), foveal avascular zone (FAZ) area, microaneurysm (MA) count, and vessel parameters in the superficial capillary plexus (SCP) and deep capillary plexus (DCP). **Results**: The SMPL group showed a greater relative reduction in FAZ area in the SCP at 3 months (3.21 ± 13.03% vs. −5.73 ± 13.3%; *p* = 0.032) with a trend toward significance at 12 months (2.37 ± 12.47% vs. −3.3 ± 7.92%; *p* = 0.086) compared to the sham group. No changes in FAZ size, MA count, and other microvascular parameters were observed in either group. In the SMPL group, BCVA improved at 3 months (T2 > T1, *p* = 0.003); CRT decreased at 12 months (T2 > T3, *p* = 0.023); MT decreased at 12 months (T2 > T3, *p* = 0.006) and MV decreased at 12 months (T2 > T3, *p* = 0.007). No changes were detected in the sham group. No treatment-related complications occurred. **Conclusions**: SMPL may improve the macular microvasculature in mild DME by reducing FAZ size in the SCP. It provides visual and functional benefits while maintaining a favorable safety profile.

## 1. Introduction

Diabetic macular edema (DME) is one of the leading causes of vision loss in patients, affecting the working-age population worldwide [1]. The prevalence of DME has been steadily increasing, mirroring the global increase in the prevalence of diabetes mellitus (DM). A prospective, population-based study by Romero-Aroca et al. reported that DME develops as a complication in 8.46% of individuals with type 1 DM and 6.36% of those with type 2 DM [2].

Subthreshold micropulse laser (SMPL) is an effective and safe therapeutic option that targets the retinal pigment epithelium (RPE). Unlike conventional lasers, SMPL stimulates biological repair mechanisms without causing visible retinal damage or chorioretinal scarring. Its effects typically manifest more slowly, becoming evident around three months after treatment, but they provide a longer-lasting impact on the retina [3]. SMPL emits energy in short, repetitive pulses, allowing the tissue to cool down between pulses, thereby avoiding thermal burns. The duty cycle (DC), which represents the effective duration of laser emission, is usually set to 5%. This means that each pulse lasts 200 μs, followed by an 1800 μs interval without energy transmission. This pulsed energy delivery enables precise, targeted treatment while minimizing damage. By preserving retinal integrity, this tissue-sparing approach helps maintain retinal function [4]. SMPL has been shown to be effective and well-tolerated, with no reported adverse events, in cases of mild to moderate macular edema with retinal thickness below 400 μm and good visual acuity [5].

Intravitreal anti-vascular endothelial growth factor (anti-VEGF) therapy has emerged as the first-line treatment for DME with vision impairment due to its superior efficacy in reducing macular edema and improving visual acuity. However, anti-VEGF agents have a rapid onset of action but a relatively short duration, necessitating frequent injections and a strict treatment regimen [6]. Several studies have demonstrated that combining SMPL with anti-VEGF agents significantly reduces the number of required injections, while achieving comparable improvements in best-corrected visual acuity (BCVA) and foveal thickness [7,8,9,10,11,12].

For cases where anti-VEGF therapy is insufficient or contraindicated, intravitreal steroid therapy is considered a second-line option. To individualize the treatment of DME, the long-acting dexamethasone implant should be considered as an initial choice in eyes with pronounced inflammatory biomarkers, prior to phacoemulsification surgery, or in patients with insufficient compliance [13].

Findings from the DRCR.net Protocol I and Protocol S studies have established that conventional laser therapy is inferior to intravitreal injections in the treatment of DME [14]. Laser photocoagulation was proven to stabilize visual acuity, but it has several adverse events, including central loss of perimetric sensitivity and potential retinal damage [15,16]. Given the limitations of conventional lasers, SMPL has gained attention as a potentially safer and effective option for mild DME with macular thickness ≤ 400 μm. Several studies have confirmed that SMPL therapy is non-inferior to conventional Early Treatment Diabetic Retinopathy Study (ETDRS) laser photocoagulation in the treatment of DME [17,18,19,20,21,22]. The DIAMOND study demonstrated that SMPL was equivalent to standard laser in effectiveness, with a slightly greater number of treatment sessions required [22].

Multimodal retinal imaging enables a comprehensive assessment and a precise approach to diagnosing and managing DME. Ultra-widefield (UWF) fundus photography, fundus autofluorescence (FAF), and optical coherence tomography (OCT) provide detailed structural visualization of the retina. Fundus fluorescein angiography (FFA) plays a crucial role in detecting retinal neovascularization, vascular leakage, capillary nonperfusion, and ischemia in both the central and peripheral retina. However, FFA is an invasive diagnostic tool, visualizing only the superficial retinal vessels, requiring an intravenous injection of fluorescent dye, and being time-consuming.

Since our study primarily focuses on DME, we employ optical coherence tomography angiography (OCTA) as a non-invasive alternative for imaging the central retinal vasculature. OCTA is an advanced three-dimensional imaging technique that enables visualization and comparison of capillaries across all retinal plexuses. It generates an OCTA map based on changes in signal strength from erythrocyte motion within retinal vessels [23]. OCTA allows for the quantification of perfused and non-perfused areas, foveal avascular zone (FAZ), and microaneurysms (MA) in individual plexuses.

The FAZ is a capillary-free region surrounding the fovea. FAZ size varies significantly among healthy individuals, which makes its evaluation and its role in macular blood supply difficult to interpret. The measured size of the FAZ in the superficial capillary plexus (SCP) and deep capillary plexus (DCP) also depends on the OCTA device used [23]. A study examining FAZ size in the SCP of healthy eyes reported a mean FAZ area of 0.329  ±  0.115  mm^2^, with a range from 0.073 mm^2^ to 0.656 mm^2^ [24]. Several studies suggest that FAZ enlargement in patients with DM correlates with the progression of diabetic retinopathy (DR), pronounced macular ischemia, and reduced visual acuity [25,26].

MA, one of the earliest signs of DR, is localized dilations of retinal capillaries due to pericyte loss and endothelial cell proliferation [27]. MA leakage contributes to the formation of DME. While FFA is commonly used to detect leaking MA, OCTA provides precise localization and quantification of MA in different capillary plexuses. The blood flow pattern within MA influences their detection on OCTA, with most MA located in the DCP [28]. Hyperreflective MA with high blood flow rates are more easily visualized, whereas hyporeflective MA with low blood flow rates may fall below the detection threshold of OCTA [29].

The aim of this study was to evaluate quantitative short- and long-term microvascular changes in OCTA following SMPL treatment in patients with DR and mild DME. The effect of SMPL on retinal vessels remains unclear. This research further investigates whether SMPL can induce regression of ischemic changes in DME or, conversely, contribute to the progression of preexisting microvascular impairments in the macula. Confirming a positive impact on macular vascular flow may help identify OCTA biomarkers of response to SMPL. The findings could contribute to the development of non-invasive OCTA technology as an additional quantitative tool for the management of early DME.

## 2. Materials and Methods

### 2.1. Study Population

This 12-month, prospective, single-masked, sham-controlled, randomized (block randomization) dual-center study of 67 eyes in 67 patients was conducted between January 2023 and December 2024. Patients were recruited from the Department of Ophthalmology at the Municipal Hospital in Rzeszów, Poland, and the Clinical Department of Ophthalmology at the Faculty of Medical Sciences in Zabrze, Medical University of Silesia in Katowice, Poland. The study followed the tenets of the Declaration of Helsinki and received approval from the Ethics Committee of the Medical University of Silesia (PCN/CBN/0052/KB1/65/I/22). All participants provided informed written consent before enrollment.

Patients were eligible for the study if they met the following criteria: (1) diagnosed with non-proliferative DR, (2) foveal thickness ≤ 325 µm, and (3) BCVA of at least 80 ETDRS letters.

### 2.2. Exclusion Criteria

The study excluded patients with any of the following: (1) foveal thickness > 325 µm, (2) BCVA below 80 ETDRS letters, (3) macular edema caused by conditions other than diabetes, including retinal vein occlusion (RVO) or age-related macular degeneration (AMD), (4) presence of vitreoretinal proliferations, predominant epiretinal membrane (ERM) or macular hole, (5) prior intravitreal administration of anti-VEGF or steroid injections within 6 months before study enrollment, (6) previous laser therapy involving the central retina, (7) vitrectomized eye, (8) age under 18 years.

### 2.3. Study Procedure

The participants were randomly divided into two groups: 33 eyes in the study group underwent SMPL treatment, while 34 eyes in the control group received sham laser therapy. No SMPL retreatment was allowed.

During baseline assessment, each patient completed a medical questionnaire. The collected demographic data included gender, height, weight, and age. Medical history was recorded, encompassing the type and duration of DM and the most recent serum glycated hemoglobin (HbA1c) level.

Following SMPL or sham treatment at baseline, participants were monitored for 12 months and underwent comprehensive ophthalmic evaluations at baseline, as well as at the 3-month and 12-month follow-up visits. These assessments included BCVA measurement, slit-lamp biomicroscopy (BQ 900, Haag-Streit Group, Köniz, Switzerland) with dilated pupil fundoscopy using a Super Field 90D lens (Volk Opticals, Mentor, OH, USA), and multimodal imaging, which included OCT, OCTA, UWF color fundus photography, and UWF FAF. These imaging techniques were employed to ensure a thorough evaluation of retinal structure and vascular integrity throughout the study.

Rescue treatment with an anti-VEGF agent was recommended for the affected eye if CRT exceeded 325 µm and BCVA fell below 75 ETDRS letters. If new neovascularization at the optic disk (NVD) or elsewhere in the retina (NVE) was identified during fundoscopy, the patient was deemed eligible for panretinal photocoagulation.

### 2.4. Ophthalmic Assessments

#### 2.4.1. BCVA Evaluation

BCVA was evaluated from a 4 m distance using a standardized Snellen chart panel (in accordance with European Standards EN ISO 8596 and EN ISO 8597) integrated with the Universal System for Eyesight Examination uSee (Medical Panel PC MPPC-203, Optopol Technology Sp. z o.o., Zawiercie, Poland). For statistical purposes, BCVA values were converted into ETDRS letter scores following the recommended calculation method [30].

#### 2.4.2. Imaging Techniques

UWF color fundus photography and FAF were conducted following pupil dilation using a high-resolution retinal imaging system (Optos California, Optos, Dunfermline, UK). Color fundus photography was utilized to evaluate the severity of DR by identifying MA, hemorrhages (H), hard exudates (HE), soft exudates (SE), and neovascularization, both NVD and NVE. The total number of MA within the central foveal area (6 mm × 6 mm grid) was manually quantified in a masked fashion by an ophthalmologist, using the multi-point tool in ImageJ Software (version 1.53c), an open-source program available at https://imagej.net/ij/ (accessed on 9 May 2025). FAF imaging was examined to detect potential retinal damage.

OCT and OCTA imaging were conducted following pupil dilation using a spectral-domain OCT system (REVO NX 130, Optopol Technology Sp. z o.o., Poland), operating at a wavelength of 850 nm with a scanning speed of 130,000 measurements per second. Each patient underwent a horizontal B-scan of the macula (7 mm × 7 mm) and a B-scan of the optic disk (6 mm × 6 mm), OCTA of the macula (6 mm × 6 mm), and OCTA of the optic disk (6 mm × 6 mm).

#### 2.4.3. OCT Evaluation

OCT image analysis was carried out using the built-in software (version 20.0.0). Automatic segmentation was applied to define the photoreceptor outer segment/RPE boundary and Bruch’s membrane. The macula was divided into nine regions based on the ETDRS grid, consisting of three concentric rings centered on the fovea, with diameters of 1 mm, 3 mm, and 6 mm. In cases of scan decentration, incorrect segmentation, or misalignment, manual adjustments were made.

Pre- and post-treatment quantitative OCT parameters were automatically assessed, including central retinal thickness (CRT), calculated as the mean thickness within the central 1 mm region; macular thickness (MT), determined as the average retinal thickness within the 6 mm ring; and macular volume (MV), measured as the total volume within the 6 mm ring.

#### 2.4.4. OCTA Evaluation

OCTA scans of the macula were recorded twice at each visit, and the higher-quality image was selected for analysis. The following boundaries were set for segmentation: (1) for the SCP, from the internal limiting membrane (ILM) to 15 µm below the inner plexiform layer (IPL); (2) for the DCP, from 15 µm below the IPL/inner nuclear layer (INL) interface to 93 µm below the IPL/INL interface. In cases of inaccurate automated segmentation, misalignment, or scan decentration, manual corrections were applied. The exclusion criteria for analysis were: (1) a quality index lower than 4/10, (2) projection artifacts, (3) motion artifacts, including white line artifacts in more than 5% of the scan section, (4) signal loss, (5) blurry images, and (6) dark areas caused by blinking.

**Figure 1:**
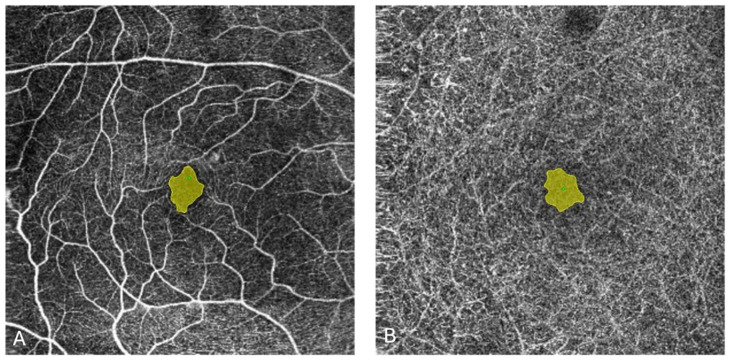
En-face OCTA image of the SCP (**A**) and DCP (**B**) of the same eye, with the automatically drawn FAZ area in yellow, as generated by the built-in software. OCTA: optical coherence tomography angiography; SCP: superficial capillary plexus; DCP: deep capillary plexus; FAZ: foveal avascular zone.

Quantitative OCTA parameters were evaluated using 6 mm × 6 mm automatic slabs at the level of the SCP and DCP. The parameters analyzed included the area of the FAZ, number of MA, vessel area density (VD), and skeleton area density (SAD). The FAZ area at the SCP and DCP levels was measured automatically (Figure 1). If an area was inaccurately detected, the contour was manually defined using the freehand selection tool, and the area was recalculated using the built-in software. The calculation of FAZ size and the quantification of the vasculature were possible using the built-in software of the OCTA device. Vascular quantification in the SCP was analyzed automatically as VD and SAD. VD was defined as the total area of perfused vasculature per unit area, while SAD represented the total area of skeletonized vasculature per unit area in the measurement region. Both VD and SAD were calculated as mean percentage values within the 6 mm ring of the ETDRS grid. Heat maps, along with values in ETDRS-specific zones, facilitated the analysis of the vascular network, represented as VD and SAD. Due to the poor quality of scans, VD and SAD were not analyzed at the DCP level. The total number of MAs in the SCP and DCP was counted manually using the multi-point tool in ImageJ Software.

Manual measurements were performed twice by a single ophthalmologist, with the mean values used as the final data for evaluation. Imaging studies that did not meet quality standards were excluded from the analysis.

Additional automated analysis was conducted using the open-source OCTAVA (OCTA Vascular Analyzer) software (version 1.0) developed by Untracht et al. [31]. The microvascular metrics provided by OCTAVA included vessel area density (VAD), vessel length density (VLD), total vessel length (TVL), mean vessel diameter (MD), median vessel diameter (MEDD), branchpoint density (BD), and mean tortuosity (MTO). We applied the recommended OCTAVA parameters for retinal OCTA image analysis [32]. Each SCP image was rescaled to 1000 × 1000 pixels. The pre-processed scan was segmented into two regions—with and without vessels—using the fuzzy thresholding method. To enhance segmentation and suppress background noise, the Frangi filter was applied with a maximum kernel size of 4 pixels. The binarized image was then automatically skeletonized using a built-in thinning algorithm. Branchpoints were identified, and vessels along with their connections were classified into different categories. Isolated elements not connected to vessels were considered noise and excluded from the analysis. The twig size control was set to 8 pixels.

The OCTA scan of the optic disk was manually evaluated for the presence of any NVD.

### 2.5. Treatment Protocol

The SMPL treatment was performed using a 577 nm true-yellow light micropulse laser (Iridex^®^ IQ 577 Laser Console, Iridex Corporation, Mountain View, CA, USA) with standard fixed parameters: spot size of 400 µm, duty cycle of 5%, exposure time of 200 ms, power of 400 mW, and application in confluent spots. The procedure was conducted under topical anesthesia (proparacaine hydrochloride 0.5% ophthalmic solution) using an Area Centralis contact lens (Volk Opticals, Mentor, OH, USA) with 1.06× magnification. Before treatment, a test burn was applied to the peripheral retina to confirm the absence of visible laser spots. The procedure was performed only if no visible reaction occurred. Laser application covered the panmacular area, including the foveal region, with no titration of laser power. The subthreshold laser spots were not visible on the central retina. The number of SMPL spots per session varied depending on macular size.

### 2.6. Statistical Analysis

Quantitative variables were analyzed using descriptive statistics, including the mean, standard deviation, median, quartiles, and minimum and maximum values. Changes in quantitative variables over time were determined by calculating the difference between baseline measurements and those recorded at 3 and 12 months post-treatment. Comparisons of quantitative variables across three repeated measurements were conducted using the Friedman test. When significant differences were identified, Wilcoxon signed-rank tests with Bonferroni correction were applied for post hoc analysis. Since the Shapiro–Wilk test indicated that the quantitative variables did not follow a normal distribution, comparisons between two groups were performed using the Mann–Whitney test.

Qualitative variables were assessed by calculating absolute frequencies and percentages. For comparisons of qualitative variables between groups, the chi-squared test was used, with Yates’ correction applied for 2 × 2 contingency tables. Fisher’s exact test was utilized for comparisons involving low expected frequencies.

Results were expressed as median with interquartile range (IQR) and mean ± standard deviation (SD). A significance level of *p* = 0.05 was applied for all statistical tests.

Statistical analyses were performed using R software (version 4.4.2).

## 3. Results

### 3.1. Baseline Characteristics

A total of 67 eyes from 67 patients were included in the baseline analysis: 33 eyes in the SMPL group and 34 eyes in the sham group.

The demographic and clinical characteristics of the participants are summarized in Table 1. The mean age of the patients was 65.42 ± 8.21 years, with no significant difference between the groups. The cohort consisted of 41.79% females and 58.21% males, with a similar sex distribution in both groups. Regarding body mass index (BMI), 16.42% of participants had a normal weight, 38.81% were overweight, and 44.78% were classified as obese, with no significant difference between the groups. The majority of participants (85.07%) had type 2 DM, while 14.93% had type 1 DM. The mean duration of DM was 19.12 ± 10.13 years, and the mean HbA1c level at baseline was 7.92 ± 1.32%, with no significant difference between the groups. These baseline characteristics indicate that the study groups were well matched.

All participants were randomly assigned to one of two groups. A total of 33 eyes (33 patients) in the study group underwent SMPL treatment, while 34 eyes (34 patients) in the control group received sham laser therapy. No additional SMPL sessions were allowed. All patients attended the 3-month follow-up examination. At the 12-month follow-up, 48 eyes were included in the analysis: 28 eyes in the study group and 20 eyes in the control group. A total of 11 eyes (1 in the SMPL group and 10 in the sham group) had been previously qualified for rescue anti-VEGF therapy. Additionally, five eyes (two in the SMPL group and three in the sham group) were withdrawn from the study, and three eyes (two in the SMPL group and one in the sham group) were excluded due to patient death. OCT and OCTA scans of insufficient quality were excluded from the analysis.

### 3.2. OCTA Parameters

**Figure 2:**
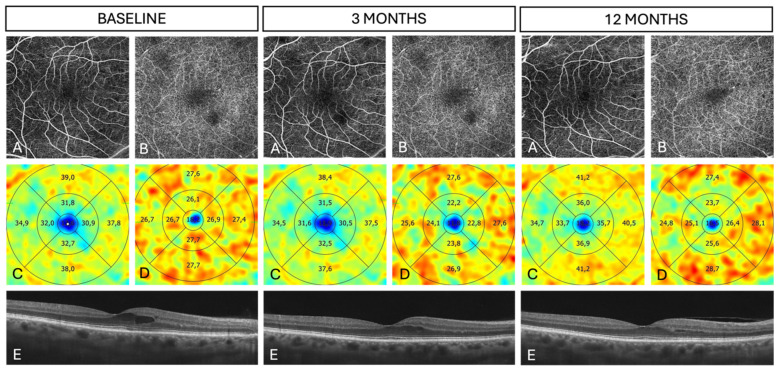
OCT and OCTA images of the right eye with mild DME at baseline (**left**), at 3 months (**middle**), and at 12 months (**right**) after SMPL treatment. (**A**) OCTA slab at the level of the SCP. (**B**) OCTA slab at the level of the DCP with artifacts from intraretinal cysts. (**C**) Heat map of the SCP showing VD in nine ETDRS zones. (**D**) Heat map of the SCP showing SAD in nine ETDRS zones. (**E**) Horizontal OCT B-scan of the macula with CRT measuring 275 µm at baseline, 263 µm at 3 months, and 254 µm at 12 months. **Note**: The software uses commas as decimal separators, and this format could not be modified. OCT: optical coherence tomography; OCTA: optical coherence tomography angiography; DME: diabetic macular edema; SMPL: subthreshold micropulse laser; SCP: superficial capillary plexus; DCP: deep capillary plexus; VD: vessel area density; ETDRS: Early Treatment Diabetic Retinopathy Study; SAD: skeleton area density; CRT: central retinal thickness.

Sample OCT and OCTA scans of a patient with DME are presented in Figure 2.

Since the patient group enrolled in the study had good BCVA and CRT within or slightly above the normal range at baseline, the changes following SMPL treatment remained subtle over time. Therefore, in addition to comparing absolute values at 3 and 12 months in the SMPL and sham groups, we also analyzed relative changes, defined as the percentage of change from the baseline value at each time point.

**Figure 3:**
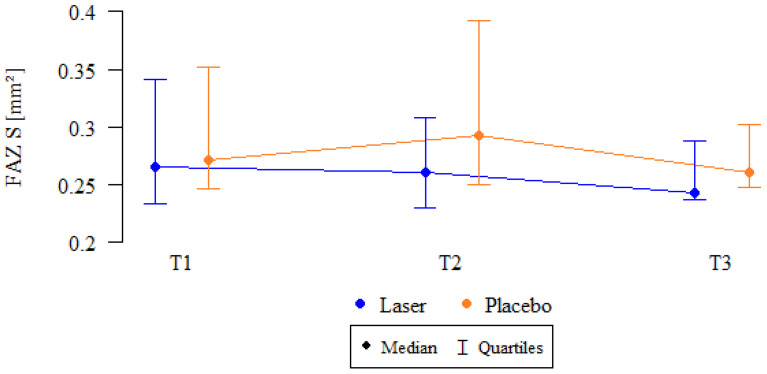
Median values of FAZ area in the superficial capillary plexus (FAZ S) over time in the SMPL and sham groups. FAZ S: foveal avascular zone in the superficial capillary plexus; SMPL: subthreshold micropulse laser; T1: baseline; T2: 3 months; T3: 12 months; Laser: SMPL group; Placebo: sham group.

Upon further analysis, the relative decrease in FAZ size in the SCP was significantly greater in the SMPL group compared to the sham group at 3 months (3.21 ± 13.03% vs. −5.73 ± 13.3%; *p* = 0.032, Mann–Whitney test). At 12 months, the relative decrease in FAZ size in the SCP was slightly above the threshold for statistical significance (2.37 ± 12.47% vs. −3.3 ± 7.92%; *p* = 0.086, Mann–Whitney test). However, no significant changes in FAZ size were observed over time in either the SCP or DCP for both the treated and control groups. Median FAZ values in the SCP are shown in Figure 3.

At all evaluated time points, the number of MA in both SCP and DCP was stable between the SMPL and sham groups, with no statistically significant differences observed.

Microvascular quantification parameters in the SCP, measured as VD and SAD, remained stable over time in both groups, with no significant differences detected. Due to the insufficient quality of OCTA scans, analysis of VD and SAD in the DCP could not be performed.

Table 2 presents the quantitative parameters assessed using built-in software with OCTA. Due to the high number of excluded OCTA scans, the table presents the number of OCTA images that were analyzed for each parameter.

The OCTA scan of the optic disk did not show the presence of any NVD.

### 3.3. OCTAVA Parameters

OCTA scans of the SCP and DCP at baseline and follow-up points were analyzed using the same preset parameters in OCTAVA software. Scans with incorrect binarization or segmentation were excluded from the analysis. Due to a high rate of errors, the analysis of DCP scans was discontinued. Ultimately, a subgroup of 17 eyes in the SMPL group and 11 eyes in the sham group was evaluated in the OCTAVA analysis of the SCP. Furthermore, as one eye from the treated group and seven eyes from the control group required rescue therapy with intravitreal anti-VEGF injections, the 12-month analysis was conducted on sixteen eyes in the SMPL group and four eyes in the sham group. Baseline values of vascular parameters and their changes over time in the SCP for both groups are shown in Table 3. Baseline parameters did not differ significantly between the SMPL and sham groups, except MTO (*p* = 0.045, Mann–Whitney test).

SMPL treatment did not lead to significant changes in the retinal microvascular network over time. Similarly, no significant differences were observed in the sham group over time.

### 3.4. OCT Parameters

A significant reduction in retinal thickness measures was observed in the SMPL group. The change in CRT was significant (*p* = 0.023, Friedman test), with a notable decrease between 3 and 12 months. The reduction in MT was also significant (*p* = 0.006), with 3-month values higher than the 12-month measurement. Likewise, the decrease in MV was significant (*p* = 0.007), with 3-month values exceeding the 12-month value. No significant changes were detected in the sham group for any of the variables.

**Figure 4:**
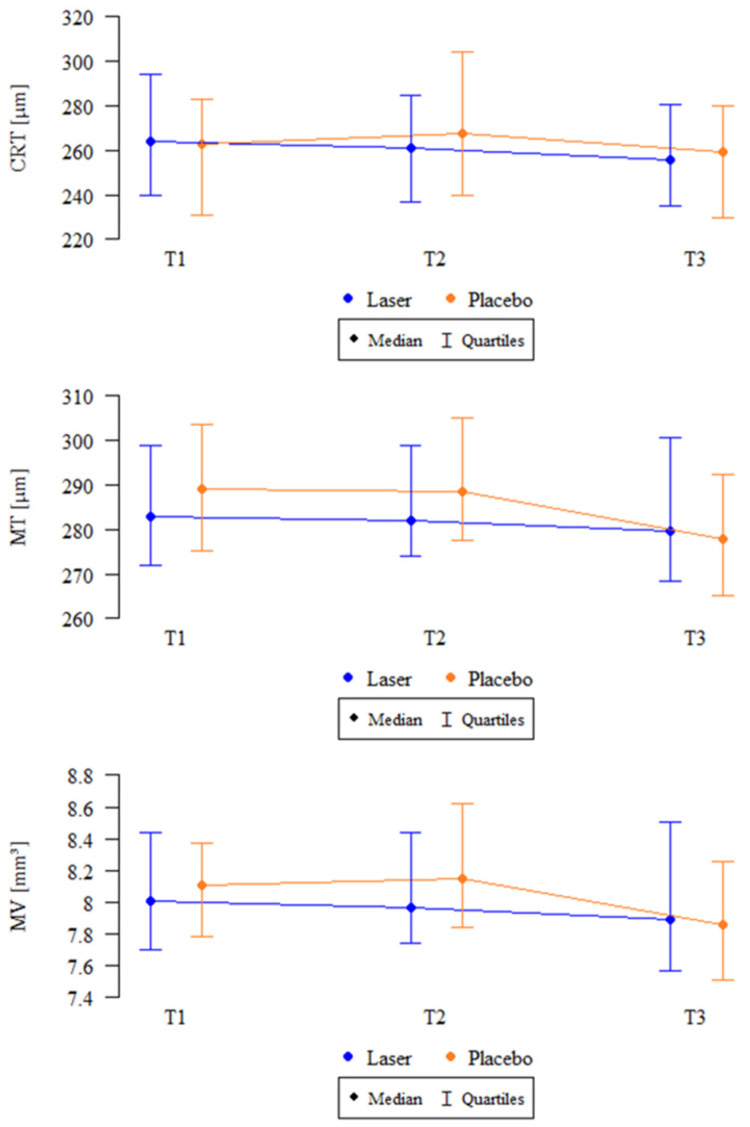
Median changes in CRT, MT, and MV over time in the SMPL and sham groups. CRT: central retinal thickness; MT: macular thickness; MV: macular volume; SMPL: subthreshold micropulse laser; T1: baseline; T2: 3 months; T3: 12 months; Laser: SMPL group; Placebo: sham group.

The anatomical parameters measured using OCT, including CRT, MT, and MV, are summarized in Table 4, and their median values are illustrated in Figure 4.

### 3.5. BCVA

BCVA improved significantly at 3 months in the SMPL group (*p* = 0.003, Friedman test). In contrast, no significant changes in BCVA were observed over time in the sham group (*p* = 0.423).

Table 5 presents BCVA values at baseline, 3 months, and 12 months for both the SMPL and sham groups.

### 3.6. MA

The number of MA counted in UWF color fundus photography within the central macular area (6 × 6 mm grid) remained stable over time in both the treated and control groups (Table 6).

### 3.7. Supplementary Analysis

In the evaluated group of 67 patients, 25 had both eyes meeting the eligibility criteria and were randomly assigned to either the SMPL or sham treatment group. To ensure statistical independence, we conducted the primary analysis using one randomly selected eye per subject. Additionally, in the Supplementary Materials, we present the results of an extended analysis including all 92 eyes from the 67 patients, which were consistent with the primary findings.

### 3.8. Safety

No treatment-related complications or adverse effects were noted at any follow-up visit. OCT scans confirmed the preservation of the ellipsoid zone (EZ) integrity over time, with no evidence of ERM formation or retinal atrophy. Additionally, FAF imaging showed no signs of RPE damage from SMPL treatment.

## 4. Discussion

Our study demonstrated the impact of SMPL on the macular vascular network, which was observed to be a relative reduction in the FAZ area within the SCP. In addition to the analysis of microvascular parameters using OCTA, supporting evaluations confirmed the efficacy and safety of SMPL in the treatment of mild DME.

The therapeutic value of SMPL in the treatment of DME has been confirmed over the years in numerous studies. However, reports assessing the macular microvascular network using OCTA remain limited to only a few publications.

The first published study evaluating quantitative parameters in OCTA (3 × 3 mm scan) following 577 nm SMPL treatment by Vujosevic et al. reported a significant reduction in FAZ size in the DCP, as well as a decrease in MA count in both the SCP and DCP at 6 months after SMPL therapy in DME. The changes in the DCP were more pronounced than in the SCP and appeared earlier in the course of treatment. No significant changes were detected in perfusion density or vessel density in the SCP and DCP. If needed, retreatment was performed at the 3-month follow-up, with 25 out of 35 eyes requiring SMPL at that time [33,34].

A subsequent study analyzing retinal capillary and choriocapillary vascular layers in a 6 × 6 mm OCTA scan compared the effects of conventional laser therapy and SMPL with a wavelength of 577 nm. Both groups demonstrated a significant improvement in microvascular perfusion in the DCP and choriocapillary plexus, reflected by increases in vessel density, vessel length, and fractal dimension at 6 months post-treatment. However, no significant changes were observed in FAZ size or MA count in either the SCP or DCP. The report does not include information regarding retreatment [35].

A research group led by Kikushima compared red SMPL (670 nm wavelength) with intravitreal anti-VEGF injections. Their findings indicated that SMPL led to an increase in vessel density exclusively in the nasal sector of the SCP and DCP at 12 months post-treatment in 3 × 3 mm OCTA slabs. Nevertheless, SMPL had no significant effect on FAZ size, and its impact on MA count was not investigated. In the SMPL group, the mean number of retreatment sessions during the one-year follow-up was 4.8 ± 1.4 [36].

A PubMed database search revealed only three published reports assessing the impact of SMPL on OCTA parameters, as referenced above. Study results are inconsistent regarding changes in FAZ size, MA count, and microvascular circulation parameters. The analyses were conducted on small patient groups, utilizing lasers with wavelengths of either 577 nm or 670 nm, and differed in treatment protocols and follow-up time points.

The findings from our study partially differ from those reported in the literature. However, a consistent observation across both previous studies and our own results is the influence of SMPL on the macular vasculature, although this effect is expressed in different ways. When comparing longitudinal changes between groups, we observed a decrease in FAZ size at 3 months in the SMPL group compared to the control group. No other significant changes were noted in the FAZ area, MA count, or vascular flow parameters.

Due to the slower blood flow rate in some MA, up to 41.0 ± 16.1% of MA may remain visible only in FFA [37]. MA plays a crucial role in vascular leakage and can serve as an indicators of DME progression. SMPL, in contrast to conventional laser treatment, does not coagulate and close MA. Despite the lack of significant differences in MA count in both the SCP and DCP, as well as in MA detected on UWF color fundus photography, a significant reduction in retinal thickness over time was observed in the SMPL group, as reflected by CRT, MV, and MT measurements. These findings support the hypothesis that the mechanism of action of SMPL differs from that of conventional laser therapy, suggesting that its therapeutic effect is primarily mediated through its impact on the RPE, rather than direct coagulation of MA.

OCTA slabs covering the macular region (6 × 6 mm) were used for the analysis. Due to the presence of artifacts (motion and projection artifacts, distortion, low signal strength) in the DCP, which affected measurement reliability, vascular parameter analysis was conducted exclusively in the SCP. Previous authors agree that vascular changes in the DCP are more pronounced than in the SCP. However, due to image quality limitations, microvascular parameters in our study were analyzed only in the SCP, and it is possible that significant differences may have been present in the DCP.

### 4.1. OCTAVA

External OCTAVA software was used to expand the OCTA analysis with an additional evaluation of the macular vascular network. Currently, there is no standardized image grading, set of metrics, or interpretation method across OCTA devices from different manufacturers, leading to variability in results depending on the device used. The goal of OCTAVA is to enable en-face image analysis across different OCTA platforms, providing a universal approach to OCTA image assessment. An updated open-source version of OCTAVA (version 2.0) is now available, introducing the capability to assess vascular parameters in nine subregions (either using a custom grid or the ETDRS grid) and incorporating additional FAZ metrics. However, our analysis began before the release of OCTAVA (version 2.0) and was conducted using OCTAVA (version 1.0). Despite this, the study followed the standard retinal image processing protocol recommended by the software developers [32].

According to the definition, in both the Revo NX 130 built-in software and the OCTAVA software, the vessel area density parameter represents the total area of perfused vasculature per unit area in the measurement region. Meanwhile, the skeleton area density in Revo NX 130 corresponds to vessel length density in OCTAVA, which defines the total area of skeletonized vasculature per unit area in the measurement region. Since significant differences (*p* < 0.001, Wilcoxon paired test) were observed between metrics generated by Revo NX 130 and OCTAVA, both in our study and in the previously cited literature, these parameters were treated as separate values. In this paper, we use the following abbreviations: VD and SAD for parameters measured using Revo NX 130, and VAD and VLD for those obtained from OCTAVA. Differences in OCTA vascular parameters result from the analysis algorithm, as well as other factors such as thresholding, filtering, and binarization methods, scan size, and acquisition pattern.

Due to insufficient image quality, projection artifacts, and low signal strength, the OCT images of the SCP were analyzed only in 28 eyes in OCTAVA software, while scans of the DCP were excluded from the analysis. Our study did not reveal a significant difference in OCTAVA-derived parameters before and after SMPL treatment. As a result, OCTAVA analysis did not prove to be a useful tool for monitoring vascular changes during SMPL treatment of DME. It is possible that using a smaller scan size (3 × 3 mm instead of 6 × 6 mm) and increasing the sample size could enhance the detection of microvascular alterations and discover additional relationships.

There are also studies examining the impact of anti-VEGF injections on vascular parameters in DME treatment, as evaluated by OCTA. Similarly to research on SMPL, the findings remain inconclusive. Some authors have reported no significant effect of anti-VEGF injections on the retinal microvascular structure [38,39,40,41]. Massengill demonstrated a decrease in vessel diameter in both the SCP and DCP, along with an improvement in FAZ circularity in the SCP after anti-VEGF treatment [42]. In another study, a notable decrease in both FAZ size and the number of MA in the SCP and DCP was observed after the therapy [43].

Patients included in our study were diagnosed with mild DME and good visual acuity. Although changes over time in the number of letters read on the ETDRS chart and central retinal measurements were relatively slight, the SMPL group demonstrated a significant improvement in BCVA, CRT, MT, and MV at 12 months. During the study, no adverse events were reported, nor was any damage detected in FAF. In all patients, OCT scans showed that the EZ remained intact, with no ERM formation following SMPL.

This study confirmed the effectiveness of SMPL in the treatment of DME after just one session. Both anatomical and functional improvements were observed over time. Additionally, SMPL demonstrated a high safety profile, with no reported side effects, making it a valuable therapeutic option for early DME management.

### 4.2. Limitations

Despite the promising results, this study has several limitations. First, the sample size was relatively small, and the study did not include a long-term follow-up beyond 12 months, which may have reduced the detection of vascular changes following SMPL treatment. A larger cohort could provide a more comprehensive understanding of the therapy’s effects. Second, OCTA imaging was limited to 6 × 6 mm scans, which may have reduced the sensitivity in detecting microvascular alterations. Third, OCTA scans of the DCP were partially excluded from the analysis. Given that previous studies suggest that vascular changes in the DCP are more pronounced than in the SCP, the lack of DCP analysis may have affected the ability to fully assess microvascular alterations. Regardless of these limitations, our findings support the efficacy and safety of SMPL in mild DME treatment and highlight the need for further research in the OCTA area.

## 5. Conclusions

In conclusion, SMPL may improve the macular microvascular network in patients with good BCVA and early-stage DME, while providing both visual and retinal structural benefits. A reduction in FAZ size in the SCP was observed in the SMPL group compared to the control group at 3-month follow-up. FAZ enlargement is recognized as a marker of progression in DME, DR, and macular ischemia. However, long-term randomized clinical trials using enhanced imaging techniques are needed to further clarify the role of OCTA-derived parameters and better understand microvascular changes in the management of mild DME.

## Figures and Tables

**Table 1:** Baseline characteristics of patients in the SMPL and sham groups (%).

Parameter		SMPL n = 33	Sham n = 34	Total n = 67	*p*
Age [years]	Mean ± SD	63.88 ± 7.72	66.91 ± 8.51	65.42 ± 8.21	*p* = 0.085
	Median (IQR)	63 (59–70)	67 (61.25–72)	66 (59.5–71)	
Sex	Female	14 (42.42%)	14 (41.18%)	28 (41.79%)	*p* = 1
	Male	19 (57.58%)	20 (58.82%)	39 (58.21%)	
BMI	Normal weight	4 (12.12%)	7 (20.59%)	11 (16.42%)	*p* = 0.62
	Overweight	14 (42.42%)	12 (35.29%)	26 (38.81%)	
	Obesity	15 (45.45%)	15 (44.12%)	30 (44.78%)	
DM type	Type 1	2 (6.06%)	8 (23.53%)	10 (14.93%)	*p* = 0.083
	Type 2	31 (93.94%)	26 (76.47%)	57 (85.07%)	
DM duration [years]	Mean ± SD	17.42 ± 9.55	20.76 ± 10.55	19.12 ± 10.13	*p* = 0.201
	Median (IQR)	15 (10–23)	20 (11.25–27)	20 (10–26.5)	
HbA1c [%]	Mean ± SD	8.05 ± 1.55	7.8 ± 1.06	7.92 ± 1.32	*p* = 0.754
	Median (IQR)	7.9 (6.9–9)	7.8 (7.2–8.6)	7.8 (6.9–8.9)	

SMPL: subthreshold micropulse laser; n: sample size; SD: standard deviation; IQR: interquartile range; BMI: body mass index; DM: diabetes mellitus; HbA1c: glycated hemoglobin; *p*, *p*-value (qualitative variables: chi-squared or Fisher’s exact test; quantitative variables: Mann–Whitney test).

**Table 2:** Mean and median values of OCTA parameters at baseline, 3 months, and 12 months in the SMPL and sham groups. Mean values are presented as ± SD; median values are shown with IQR in brackets.

Parameter	Group	n	Baseline	n	3 Months	n	12 Months	*p*
FAZ SCP (µm^2^)	SMPL	31/33	298 ± 85	30/33	283 ± 83	26/28	276 ± 79	0.435
			266 (233–342)		260 (230–309)		243 (238–288)	
	Sham	31/34	332 ± 211	32/34	351 ± 209	19/20	324 ± 239	0.494
			271 (246–352)		293 (250–392)		261 (248–302)	
FAZ DCP (µm^2^)	SMPL	24/33	393 ± 140	25/33	393 ± 146	22/28	366 ± 96	0.182
			386 (293–441)		344 (282–446)		340 (308–398)	
	Sham	25/34	434 ± 241	25/34	456 ± 258	12/20	442 ± 340	0.196
			384 (331–446)		406 (342–457)		346 (310–395)	
MA SCP	SMPL	31/33	2.87 ± 2.54	32/33	3.25 ± 4.03	27/28	2.93 ± 2.32	0.697
			2 (1–4)		2 (1–3.25)		2 (1–4)	
	Sham	33/34	3.64 ± 3.4	33/34	3.97 ± 3.42	20/20	3 ± 2.10	0.157
			3 (1–4)		3 (1–5)		2 (1–5)	
MA DCP	SMPL	31/33	9.29 ± 9.02	32/33	8.97 ± 9.03	26/28	9.04 ± 9.37	0.53
			7 (3–11.5)		5.5 (3–9.25)		4.5 (3.25–13)	
	Sham	32/34	9.41 ± 9.15	32/34	10.12 ± 9.76	18/20	7.61 ± 7.06	0.684
			4.5 (3–14.5)		6 (3–14.75)		4.5 (3–9)	
VD SCP (%)	SMPL	27/33	36.68 ± 1.66	26/33	36.03 ± 1.66	22/28	35.73 ± 2.01	0.167
			37 (34.95–37.6)		36 (35.02–36.62)		36.25 (34.9–37.27)	
	Sham	24/34	34.94 ± 2.04	25/34	34.22 ±2.65	14/20	34.81 ± 2.54	0.368
			34.95 (33.3–36.7)		34.6 (32.1–36.2)		35 (33.3–36.45)	
SAD SCP (%)	SMPL	27/33	25.89 ± 1.21	26/33	25.74 ± 1.85	22/28	25.4 ± 1.56	0.842
			26 (25.15–26.8)		25.75 (24.85–26.45)		25.8 (25.15–26.3)	
	Sham	24/34	24.92 ± 2.89	25/34	24.25 ± 1.7	14/20	24.36 ± 1.76	0.495
			24.2 (23.58–25.88)		24.7 (22.6–25.2)		24.7 (22.68–25.77)	

OCTA: optical coherence tomography angiography; SMPL: subthreshold micropulse laser; SD, standard deviation; IQR, interquartile range; n, number of eyes analyzed; FAZ, foveal avascular zone; SCP, superficial capillary plexus; DCP, deep capillary plexus; MA, microaneurysms; VD, vessel area density; SAD, skeletonized area density; *p*, *p*-value (Friedman test).

**Table 3:** Baseline values and longitudinal changes in vascular parameters of the SCP in the OCTAVA analyzer. Mean values are presented with ± SD, median values with IQR in brackets.

Parameter	Group	Baseline Values			Change in 3 Months		Change in 12 Months		
		Mean	Median	*p*	Mean	*p*		Mean	*p*
VAD (%)	SMPL	21.71 ± 2.26	21 (20–23)	0.223	0.71 ± 1.9	0.866		−0.56 ± 1.55	0.961
	Sham	20.55 ± 1.63	21 (19.5–21)		0.64 ± 1.21			−0.5 ± 1.29	
VLD (%)	SMPL	2.35 ± 0.25	2.3 (2.14–2.42)	0.346	0.06 ± 0.18	0.925		−0.05 ± 0.19	0.704
	Sham	2.27 ± 0.17	2.28 (2.14–2.37)		0.06 ± 0.14			−0.08 ± 0.14	
TVL (mm)	SMPL	23.49 ± 2.55	22.98 (21.4–24.24)	0.329	0.47 ± 1.8	0.832		−1.66 ± 5.6	0.682
	Sham	21.81 ± 3.95	22.8 (21.26–23.66)		1.53 ± 4.24			−0.8 ± 1.35	
MD (µm)	SMPL	5.12 ± 0.33	5 (5–5)	0.67	0 ± 0.35	0.597		0.12 ± 0.5	0.635
	Sham	5.18 ± 0.4	5 (5–5)		0.09 ± 0.54			0 ± 0	
MEDD (µm)	SMPL	5.29 ± 0.47	5 (5–6)	0.411	0.12 ± 0.6	0.515		0.38 ± 1.36	0.747
	Sham	5.45 ± 0.52	5 (5–6)		0.27 ± 0.65			0 ± 0.82	
BD (nodes/mm)	SMPL	10.79 ± 1.64	10.6 (10.11–11.64)	0.384	−0.51 ± 2.75	0.746		−0.69 ± 1.98	1
	Sham	9.51 ± 2.85	10.6 (9.55–10.77)		0.82 ± 2.8			−0.13 ± 0.17	
MTO	SMPL	0.12 ± 0.01	0.12 (0.11–0.12)	0.045 *	0 ± 0.01	0.334		0 ± 0.01	0.578
	Sham	0.12 ± 0.01	0.13 (0.12–0.13)		0 ± 0.01			0 ± 0	

SCP: superficial capillary plexus; SD: standard deviation; IQR: interquartile range; SMPL: subthreshold micropulse laser; VAD: vessel area density; VLD: vessel length density; TVL: total vessel length; MD: mean vessel diameter; MEDD: median vessel diameter; BD: branchpoint density; MTO: mean tortuosity; *p*: *p*-value (Mann–Whitney test); * statistically significant (*p* < 0.05).

**Table 4:** Changes in anatomical parameters over time measured by OCT. Mean values are presented with ± SD, median values with IQR in brackets.

Parameter	Group	N	Baseline	n	3 Months	n	12 Months	*p*
CRT (µm)	SMPL	33	264.76 ± 31.11 264 (240–294)	33	259.91 ± 28.33 261 (237–285)	28	256.32 ± 30.90 255.5 (235–280.5)	0.023 * T2 > T3
	Sham	34	261.41 ± 33.9 263 (231–283)	34	276.32 ± 51.17 267.5 (240–304.5)	20	254.5 ± 34.16 259.5 (229.75–280)	0.669
MT (µm)	SMPL	33	287.27 ± 19.02 283 (272–299)	33	287.33 ± 20.55 282 (274–299)	28	285 ± 22.28 279.5 (268.25–300.75)	0.006 * T2 > T3
	Sham	34	287.97 ± 21.62 289 (275.25–303.5)	34	292.04 ± 26.87 288.5 (277.5–305)	20	282.65 ± 28.6 278 (265.25–292.5)	0.238
MV (mm^2^)	SMPL	33	8.12 ± 0.54 8.01 (7.7–8.44)	33	8.12 ± 0.58 7.97 (7.74–8.44)	28	8.06 ± 0.63 7.89 (7.57–8.51)	0.007 * T2 > T3
	Sham	34	8.12 ± 0.61 8.11 (7.78–8.38)	34	8.26 ± 0.76 8.14 (7.84–8.62)	20	7.99 ± 0.81 7.86 (7.51–8.26)	0.311

OCT: optical coherence tomography; SD: standard deviation; IQR: interquartile range; CRT: central retinal thickness; MT: macular thickness; MV: macular volume; SMPL: subthreshold micropulse laser; T1: baseline; T2: 3 months; T3: 12 months; *p*: *p*-value (Friedman test + post hoc Wilcoxon paired tests with Bonferroni correction); * statistically significant (*p* < 0.05).

**Table 5:** Changes in BCVA (ETDRS letter score) over time in the SMPL and sham groups.

Group	Time	n	Mean ± SD	Median (IQR)	*p*
SMPL	Baseline	33	82.97 ± 2.16	83 (81–85)	*p* = 0.003 * T2 > T1
	3 months	33	83.3 ± 4.74	85 (83–85)	
	12 months	28	83.5 ± 4.06	85 (83–85)	
Sham	Baseline	34	82.88 ± 2.21	83 (80–85)	*p* = 0.423
	3 months	34	81.82 ± 2.98	82.5 (80–84.75)	
	12 months	20	82.20 ± 3.62	84 (80–85)	

BCVA: best-corrected visual acuity; ETDRS: Early Treatment Diabetic Retinopathy Study; SMPL: subthreshold micropulse laser; SD: standard deviation; IQR: interquartile range; T1: baseline; T2: 3 months; *p*: *p*-value (Friedman test + post hoc Wilcoxon paired tests with Bonferroni correction); * statistically significant (*p* < 0.05).

**Table 6:** Changes in the number of MA in the central macular area assessed using UWF color fundus photography over time in the SMPL and sham groups.

Group	Time	n	Mean ± SD	Median (IQR)	*p*
SMPL	Baseline	33	9.21 ± 11.70	5 (1–13)	*p* = 0.96
	3 months	33	9.36 ± 12.81	4 (2–12)	
	12 months	28	9.5 ± 11.03	4.5 (2–12.75)	
Sham	Baseline	33	10.97 ± 15.79	4 (2–11)	*p* = 0.721
	3 months	34	14.06 ± 18.59	6 (1.25–17.25)	
	12 months	20	8.05 ± 11.98	3 (2–7)	

MA: microaneurysms; UWF: ultra-widefield; SMPL: subthreshold micropulse laser; SD: standard deviation; IQR: interquartile range; n: number of eyes; *p*: *p*-value (Friedman test).

## Data Availability

The data supporting the reported results are available from the corresponding author upon reasonable request.

## Supplementary Materials

The following supporting information can be downloaded at: https://www.mdpi.com/article/10.3390/biomedicines13051194/s1, Figure S1: Median values of FAZ area in the superficial capillary plexus (FAZ S) over time in the SMPL and sham groups; Figure S2: Median changes in CRT, MT, and MV over time in the SMPL and sham groups; Table S1: Baseline characteristics of patients in the SMPL and sham groups (%); Table S2: Mean and median values of OCTA parameters at baseline, 3 months, and 12 months in the SMPL and sham groups; Table S3: Baseline values and longitudinal changes in vascular parameters of the SCP in the OCTAVA analyzer; Table S4: Changes in anatomical parameters over time measured by OCT; Table S5: Changes in BCVA (ETDRS letter score) over time in the SMPL and sham groups; Table S6: Changes in the number of MA in the central macular area assessed using UWF color fundus photography over time in the SMPL and sham groups.

## Abbreviations

The following abbreviations are used in this manuscript:

DME	Diabetic macular edema
DM	Diabetes mellitus
SMPL	Subthreshold micropulse laser
RPE	Retinal pigment epithelium
DC	Duty cycle
VEGF	Vascular endothelial growth factor
BCVA	Best-corrected visual acuity
ETDRS	Early Treatment Diabetic Retinopathy Study
UWF	Ultra-widefield
FAF	Fundus autofluorescence
OCT	Optical coherence tomography
FFA	Fundus fluorescein angiography
OCTA	Optical coherence tomography angiography
FAZ	Foveal avascular zone
MA	Microaneurysms
SCP	Superficial capillary plexus
DCP	Deep capillary plexus
DR	Diabetic retinopathy
RVO	Retinal vein occlusion
AMD	Age-related macular degeneration
ERM	Epiretinal membrane
HbA1c	Glycated hemoglobin
NVD	Neovascularization at the optic disk
NVE	Neovascularization elsewhere in the retina
H	Hemorrhages
HE	Hard exudates
SE	Soft exudates
CRT	Central retinal thickness
MT	Macular thickness
MV	Macular volume
ILM	Internal limiting membrane
IPL	Inner plexiform layer
INL	Inner nuclear layer
VD	Vessel area density
SAD	Skeleton area density
VAD	Vessel area density
VLD	Vessel length density
TVL	Total vessel length
MD	Mean vessel diameter
MEDD	Median vessel diameter
BD	Branchpoint density
MTO	Mean tortuosity
IQR	Interquartile range
SD	Standard deviation
BMI	Body mass index
EZ	Ellipsoid zone
