# Supplementary material for: Impact of Subthreshold Micropulse Laser on the Vascular Network in Diabetic Macular Edema: An Optical Coherence Tomography Angiography Study
Source: Biomedicines. 2025 May 14;13(5):1194. doi: 10.3390/biomedicines13051194 (PMC12109209; doi:10.3390/biomedicines13051194)
Supplement: Supplementary file 1 [file biomedicines-13-01194-s001.zip › biomedicines-3597021-supplementary.pdf]

Impact of Subthreshold Micropulse Laser on the Vascular Network in Diabetic Macular Edema: An Optical Coherence Tomography Angiography Study

Supplementary Analysis: Inclusion of 92 Eligible Eyes from 67 Patients

While the main analysis adheres to the standard practice of including one eye per patient to maintain statistical independence, we conducted an additional supplementary analysis that included all 92 eligible eyes from 67 patients (42 patients contributed one eye, and 25 contributed both eyes).

This extended analysis was performed for several reasons. First, the frequent occurrence of artifacts in OCTA imaging and the exclusion of one eye per patient reduced the sample size, potentially compromising statistical power. Including both eyes helps to address this limitation. Second, in many cases, the two eyes presented with distinct morphological characteristics of DME, such as differences in edema location and severity. This variability indicates that each eye provided unique data, justifying their separate inclusion in the analysis. Finally, from a clinical standpoint, both eyes are often treated independently in real-world practice. Therefore, analyzing both eyes more accurately reflects routine clinical scenarios and enhances the applicability of our findings.

The results were consistent with those obtained from the single-eye analysis. As in the main analysis, the supplementary analysis confirmed statistically significant changes in the same parameters: FAZ size in the SCP, CRT, MT, MV, and BCVA. The relative reduction in FAZ size in the SCP was significantly greater in the SMPL group compared to the sham group, not only at 3 months but also at 12 months. The results are discussed in detail in the text below.

Results

1. Baseline Characteristics

A total of 92 eyes from 67 patients were included in the baseline analysis. Among them, 25 patients had both eyes enrolled, while 42 patients had only one eye included in the study. This supplement presents results from all patients, regardless of whether one or both eyes were recruited.

The demographic and clinical characteristics of the participants are summarized in Table S1. The mean age of the patients was 65.02 ± 7.99 years, with no significant difference between the groups. The study cohort consisted of 40.22% females and 59.78% males, with a similar sex distribution in both groups. Regarding body mass index (BMI), 14.13% of participants had a normal weight, 39.13% were overweight, and 46.74% were classified as obese, with no significant difference between the groups. The majority of participants (86.96%) had type 2 DM, while 13.04% had type 1 DM. The mean duration of DM was 19.05 ± 9.98 years, which was significantly longer in the sham group (21.2 ± 10.09 years) compared to the SMPL group (16.39 ± 9.29 years). The mean HbA1c level at baseline was 8.00 ± 1.40%, with no significant difference between the groups. These baseline characteristics indicate that the study groups were well matched, except for a longer duration of DM in the sham group.

Table S1. Baseline characteristics of patients in the SMPL and sham groups (%).

Parameter		SMPL n=41	Sham n=51	Total n=92	p
Age [years]	Mean ± SD	64.27±7.11	65.63±8.66	65.02±7.99	p=0.283
	Median (IQR)	63 (60-69)	66 (60-71.5)	65.5 (60-71)	
Sex	Female	15 (36.59%)	22 (43.14%)	37 (40.22%)	p=0.672
	Male	26 (63.41%)	29 (56.86%)	55 (59.78%)	

Parameter		SMPL n=41	Sham n=51	Total n=92	p
BMI	Normal weight	4 (9.76%)	9 (17.65%)	13 (14.13%)	p=0.348
	Overweight	19 (46.34%)	17 (33.33%)	36 (39.13%)	
	Obesity	18 (43.90%)	25 (49.02%)	43 (46.74%)	
DM type	Type 1	2 (4.88%)	10 (19.61%)	12 (13.04%)	p=0.076
	Type 2	39 (95.12%)	41 (80.39%)	80 (86.96%)	
DM duration [years]	Mean ± SD	16.39±9.29	21.2±10.09	19.05±9.98	p=0.031 *
	Median (IQR)	15 (10-20)	20 (11.5-29)	19.5 (10-27)	
HbA1c [%]	Mean ± SD	8.02±1.49	7.98±1.34	8±1.4	p=0.912
	Median (IQR)	7.5 (6.9-8.9)	8 (7.15-8.9)	7.9 (6.9-8.9)	

SMPL, subthreshold micropulse laser; n, sample size; SD, standard deviation; IQR, interquartile range; BMI, body mass index; DM, diabetes mellitus; HbA1c, glycated hemoglobin; p, p-value (qualitative variables: chi-squared or Fisher's exact test, quantitative variables: Mann-Whitney test); * statistically significant ($p < 0.05$).

All participants were randomly assigned to one of two groups. A total of 41 eyes (37 patients) in the study group underwent SMPL treatment, while 51 eyes (46 patients) in the control group received sham laser therapy. No additional SMPL sessions were allowed. All patients attended the 3-month follow-up examination. At the 12-month follow-up, 66 eyes were included in the analysis: 33 in the study group and 33 in the control group. A total of 14 eyes had been previously qualified for rescue anti-VEGF therapy, 8 eyes were withdrawn from the study, and 4 eyes were excluded due to patient death. OCT and OCTA scans of insufficient quality were excluded from the analysis.

2. OCTA Parameters

Since the patient group enrolled in the study had good BCVA and CRT within or slightly above the normal range at baseline, the changes following SMPL treatment remained subtle over time. Therefore, in addition to comparing absolute values at 3 and 12 months in the SMPL and sham groups, we also analyzed relative changes, defined as the percentage of change from the baseline value at each time point.

Upon further analysis, the relative decrease in FAZ size in the SCP was significantly greater in the SMPL group compared to the sham group at 3 months (3.03 ± 12.5% vs. -5.84 ± 13.84%; p = 0.014, Mann-Whitney test) and at 12 months (2.93 ± 11.98% vs. -4.98 ± 10.55%; p = 0.015, Mann-Whitney test). However, no significant changes in FAZ size were observed over time in either the SCP or DCP for both the treated and control groups. Median FAZ values in the SCP are shown in Figure S1.

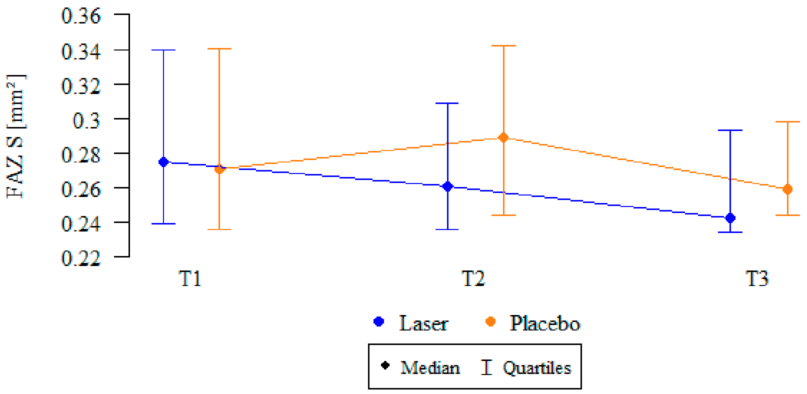

Figure S1. Median values of FAZ area in the superficial capillary plexus (FAZ S) over time in the SMPL and sham groups. FAZ S, foveal avascular zone in the superficial capillary plexus; SMPL, subthreshold micropulse laser; T1, baseline; T2, 3 months; T3, 12 months; Laser, SMPL group; Placebo, sham group.

At all evaluated time points, the number of MA in both SCP and DCP was stable between the SMPL and sham groups, with no statistically significant differences observed.

Microvascular quantification parameters in the SCP, measured as VD and SAD, remained stable over time in both groups, with no significant differences detected. Due to the insufficient quality of OCTA scans, analysis of VD and SAD in the DCP could not be performed.

Table S2 presents the quantitative parameters assessed using built-in software with OCTA. Due to the high number of excluded OCTA scans, the table presents the number of OCTA images that were analyzed for each parameter.

The OCTA scan of the optic disc did not show the presence of any NVD.

Table S2. Mean and median values of OCTA parameters at baseline, 3 months, and 12 months in the SMPL and sham groups. Mean values are presented as ± SD; median values are shown with IQR in brackets.

Parameter	Group	n	Baseline	n	3 months	n	12 months	p
FAZ SCP (μm^2)	SMPL	38/41	338±289<br>275 (239-340)	38/41	326±297<br>260 (236-309)	31/33	275±74<br>243 (234-293)	0.257
	Sham	50/51	310±172<br>270 (236-241)	47/51	325±178<br>289 (244-342)	29/33	301±195<br>259 (244-298)	0.102
FAZ DCP (μm^2)	SMPL	32/41	446±327<br>387 (304-448)	32/41	457±412<br>353 (298-440)	27/33	362±90<br>343 (308-390)	0.584
	Sham	39/51	393±204<br>364 (299-414)	38/51	415±219<br>381 (312-444)	22/33	388±256<br>335 (292-379)	0.193
MA SCP	SMPL	39/41	3.15±2.48<br>3 (1-4)	40/41	3.33±3.66<br>3 (1-4)	32/33	3±2.54<br>2 (1-4)	0.881
	Sham	51/51	3.67±3.23<br>3 (2-4)	48/51	3.79±3.36<br>3 (1-5)	29/33	2.55±1.86<br>2 (1-3)	0.173
MA DCP	SMPL	39/41	9.74±9.46<br>7 (3-13)	40/41	10.03±9.6<br>6 (3-16.25)	31/33	9.45±10.86<br>4 (3-12)	0.642
	Sham	51/51	9.85±9.94<br>6 (3-15)	47/51	10.26±10.15<br>6 (3-14.5)	27/33	9.33±10.75<br>5 (3-11)	0.913
VD SCP (%)	SMPL	33/41	36.08±2.08<br>36.8 (34.6-37.6)	31/41	35.90±2.02<br>36.0 (34.95-36.7)	27/33	35.5±1.97<br>35.7 (34.2-	0.446
	Sham	41/51	35.66±1.96<br>36.2 (34.3-37.2)	39/51	34.80 ±2.44<br>34.9 (33.65-36.6)	24/33	35.13±2.11<br>35.35 (34.5-	0.482
SAD SCP (%)	SMPL	33/41	25.49±1.47<br>25.5 (24.7-26.5)	31/41	25.57±1.88<br>25.7 (24.65-26.4)	27/33	25.24±1.58<br>25.8 (25-26.2)	0.823
	Sham	41/51	25.31±2.34<br>25.4 (24-26.4)	39/51	24.69±1.64<br>24.7 (23.85-25.8)	24/33	24.8±1.51<br>25.2 (24.23-	0.118

OCTA, optical coherence tomography angiography; SMPL, subthreshold micropulse laser; SD, standard deviation; IQR, interquartile range; n, number of eyes analyzed; FAZ, foveal avascular zone; SCP, superficial capillary plexus; DCP, deep capillary plexus; MA, microaneurysms; VD, vessel area density; SAD, skeletonized area density; p, p-value (Friedman test).

3. OCTAVA Parameters

OCTA scans of the SCP and DCP at baseline and follow-up points were analyzed using the same preset parameters in OCTAVA software. Scans with incorrect binarization or segmentation were excluded from the analysis. Due to a high rate of errors, the analysis of DCP scans was discontinued. Ultimately, a subgroup of 21 eyes from 18 patients in the SMPL group and 14 eyes from 10 patients in the sham group was evaluated in the OCTAVA analysis of the SCP. Furthermore, as 2 eyes from the

treated group and 7 eyes from the control group required rescue therapy with intravitreal anti-VEGF injections, the 12-month analysis was conducted on 19 eyes in the SMPL group and 7 eyes in the sham group. Baseline values of vascular parameters and their changes over time in the SCP for both groups are shown in Table S3. Baseline parameters did not differ significantly between the SMPL and sham groups. SMPL treatment did not lead to significant changes in the retinal microvascular network over time. Similarly, no significant differences were observed in the sham group over time.

Table S3. Baseline values and longitudinal changes in vascular parameters of the SCP in the OCTAVA analyzer. Mean values are presented with ± SD, median values with IQR in brackets.

Parameter	Group	Baseline values			Change in 3 months		Change in 12 months	
		Mean	Median	p	Mean	p	Mean	p
VAD (%)	SMPL	21.38±2.27	21 (20-23)	0.986	0.48±1.81	1	-0.47±1.58	0.612
	Sham	21.21±1.97	21 (20-22.75)		0.36±1.22		-0.86±1.21	
VLD (%)	SMPL	2.31±0.25	2.28 (2.14-2.41)	0.787	0.05±0.17	0.533	-0.04±0.18	0.234
	Sham	2.34±0.20	2.34 (2.19-2.46)		0.02±0.16		-0.11±0.14	
TVL (mm)	SMPL	23.13±2.48	22.76 (21.38-24.1)	0.907	0.36±1.69	0.711	-1.36±5.19	0.231
	Sham	22.67±3.87	23.38 (21.88-24.58)		0.9±3.94		-1.11±1.39	
MD (μm)	SMPL	5.14±0.36	5 (5-5)	1	-0.05±0.38	0.426	0.05±0.52	0.724
	Sham	5.14±0.36	5 (5-5)		0.07±0.47		0.14±0.38	
MEDD (μm)	SMPL	5.33±0.48	5 (5-6)	0.587	0.05±0.59	0.43	0.32±1.25	0.922
	Sham	5.43±0.51	5 (5-6)		0.21±0.7		0.14±0.9	
BD (nodes/mm)	SMPL	10.57±1.69	10.49 (9.27-11.64)	0.946	-0.41±2.51	0.96	-0.59±1.91	0.534
	Sham	9.9±2.62	10.69 (9.8-10.92)		0.49±2.55		-0.73±0.93	
MTO	SMPL	0.12±0.01	0.12 (0.11-0.12)	0.066	0±0.01	0.771	0±0.01	0.239
	Sham	0.12±0.01	0.12 (0.12-0.13)		0±0.01		0±0.01	

SCP, superficial capillary plexus; SD, standard deviation; IQR, interquartile range; SMPL, subthreshold micropulse laser; VAD, vessel area density; VLD, vessel length density; TVL, total vessel length; MD, mean vessel diameter; MEDD, median vessel diameter; BD, branchpoint density; MTO, mean tortuosity; p, p-value (Mann-Whitney test).

4. OCT Parameters

A significant reduction in retinal thickness measures was observed in the SMPL group. The change in CRT was significant (p = 0.044, Friedman test), with a notable decrease between 3 and 12 months. The reduction in MT was also significant (p = 0.002), with both baseline and 3-month values higher than the 12-month measurement. Likewise, the decrease in MV was significant (p = 0.002), with baseline and 3-month values exceeding the 12-month value. No significant changes were detected in the sham group for any of the variables.

The anatomical parameters measured using OCT, including CRT, MT, and MV, are summarized in Table S4, and their median values are illustrated in Figure S2.

Table S4. Changes in anatomical parameters over time measured by OCT. Mean values are presented with ± SD, median values with IQR in brackets.

Parameter	Group	n	Baseline	n	3 months	n	12 months	p
CRT (μm)	SMPL	41	264.85±28.22<br>264 (241-291)	41	266.41±31.22<br>269 (240-286)	33	258.76±29.86<br>259 (237-282)	0.044 *<br>T2>T3
	Sham	51	261.39±29.63<br>262 (234-282.5)	51	272.49±44.12<br>266 (237-292)	33	257.03±27.76<br>261 (237-278)	0.52
MT (μm)	SMPL	41	286.54±19.59<br>283 (270-299)	41	287.73±21.85<br>282 (271-302)	33	284.67±22.78<br>279 (266-299)	0.002 *<br>T2,T1>T3

	Sham	51	288.14±20.73<br>286 (276.5-306)	51	291.04±24.36<br>288 (278-305)	33	284.12±24.08<br>284 (270-297)	0.21
MV (mm²)	SMPL	41	8.11±0.55<br>8.01 (7.70-8.44)	41	8.14±0.62<br>7.97 (7.66-8.54)	33	8.05±0.65<br>7.88 (7.52-8.46)	0.002 *<br>T1,T2>T3
	Sham	51	8.13±0.58<br>8.08 (7.8-8.52)	51	8.23±0.69<br>8.13 (7.86-8.61)	33	8.03±0.68<br>8.03 (7.64-8.4)	0.214

OCT, optical coherence tomography, SD, standard deviation; IQR, interquartile range; CRT, central retinal thickness; MT, macular thickness; MV, macular volume; SMPL, subthreshold micropulse laser; T1, baseline, T2, 3 months, T3, 12 months, p, p-value (Friedman test + post-hoc Wilcoxon paired tests with Bonferroni correction); * statistically significant ($p < 0.05$).

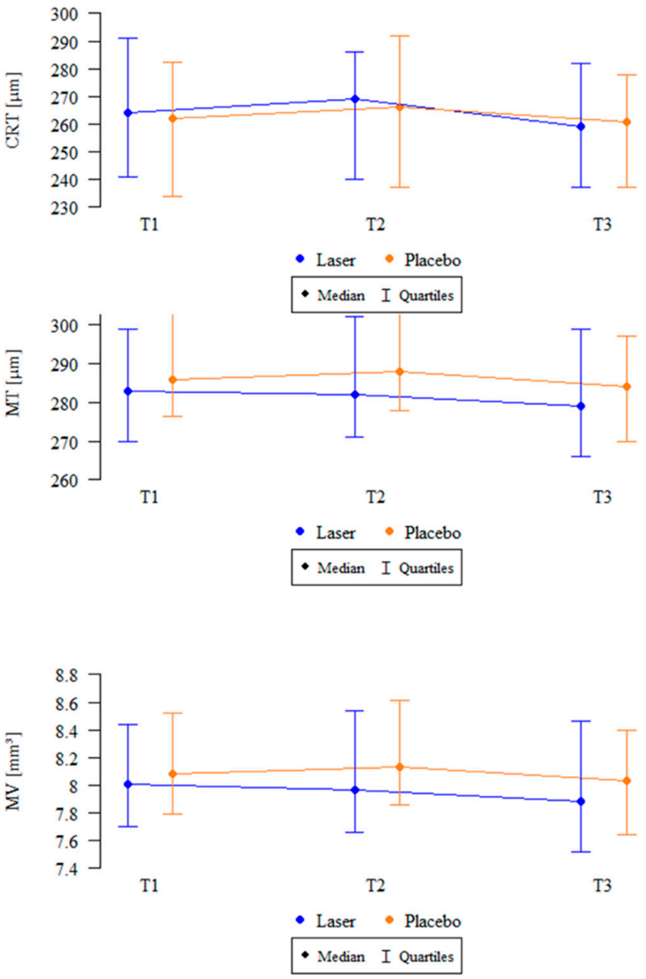

Figure S2. Median changes in CRT, MT, and MV over time in the SMPL and sham groups. CRT, central retinal thickness; MT, macular thickness; MV, macular volume; SMPL, subthreshold micropulse laser; T1, baseline; T2, 3 months; T3, 12 months; Laser, SMPL group; Placebo, sham group.

5. BCVA

At baseline, there was no significant difference in BCVA, expressed as the ETDRS letter score, between the treated and control groups (p = 0.361, Mann–Whitney test).

BCVA improved significantly at 3 months in the SMPL group (p = 0.002, Friedman test). In contrast, no significant changes in BCVA were observed over time in the sham group (p = 0.632).

Table S5 presents BCVA values at baseline, 3 months, and 12 months for both the SMPL and sham groups.

Table S5. Changes in BCVA (ETDRS letter score) over time in the SMPL and sham groups.

Group	Time	n	Mean ± SD	Median (IQR)	p
SMPL	Baseline	41	82.88±2.19	83 (81-85)	p=0.002 * T2>T1
	3 months	41	83.24±4.51	85 (83-85)	
	12 months	33	83.52±3.81	85 (83-85)	
Sham	Baseline	51	83.24±2.2	84 (81-85)	p=0.632
	3 months	51	82.41±3.14	83 (80-85)	
	12 months	33	82.7±3.35	84 (80-85)	

BCVA, best-corrected visual acuity; ETDRS, Early Treatment Diabetic Retinopathy Study; SMPL, subthreshold micropulse laser; SD, standard deviation; IQR, interquartile range; T1, baseline; T2, 3 months, p, p-value (Friedman test + post-hoc Wilcoxon paired tests with Bonferroni correction); * statistically significant ($p < 0.05$).

6. MA

The number of MA counted in UWF color fundus photography within the central macular area (6 × 6 mm grid) did not differ significantly between groups at baseline ($p = 0.718$, Mann-Whitney test) and remained stable over time in both the treated and control groups (Table S6).

Table S6. Changes in the number of MA in the central macular area assessed using UWF color fundus photography over time in the SMPL and sham groups.

Group	Time	n	Mean ± SD	Median (IQR)	p
SMPL	Baseline	41	10.85±14.23	5 (2-13)	p=0.891
	3 months	40	11.15±14.11	5.5 (2-14.5)	
	12 months	33	10.64±13.15	5 (2-14)	
Sham	Baseline	50	10.16±13.75	4 (2-14.25)	p=0.519
	3 months	51	11.63±16.02	6 (1-12)	
	12 months	33	7.88±11	3 (2-7)	

MA, microaneurysms; UWF, ultra-widefield; SMPL, subthreshold micropulse laser; SD, standard deviation; IQR, interquartile range; n, number of eyes; p, p-value (Friedman test).

7. Safety

No treatment-related complications or adverse effects were noted at any follow-up visit. OCT scans confirmed the preservation of the ellipsoid zone (EZ) integrity over time, with no evidence of ERM formation or retinal atrophy. Additionally, FAF imaging showed no signs of RPE damage from SMPL treatment.
